# Properties and Modeling of GWAS when Complex Disease Risk Is Due to Non-Complementing, Deleterious Mutations in Genes of Large Effect

**DOI:** 10.1371/journal.pgen.1003258

**Published:** 2013-02-21

**Authors:** Kevin R. Thornton, Andrew J. Foran, Anthony D. Long

**Affiliations:** Department of Ecology and Evolutionary Biology, University of California Irvine, Irvine, California, United States of America; University of Chicago and Howard Hughes Medical Institute, United States of America

## Abstract

Current genome-wide association studies (GWAS) have high power to detect intermediate frequency SNPs making modest contributions to complex disease, but they are underpowered to detect rare alleles of large effect (RALE). This has led to speculation that the bulk of variation for most complex diseases is due to RALE. One concern with existing models of RALE is that they do not make explicit assumptions about the evolution of a phenotype and its molecular basis. Rather, much of the existing literature relies on arbitrary mapping of phenotypes onto genotypes obtained either from standard population-genetic simulation tools or from non-genetic models. We introduce a novel simulation of a 100-kilobase gene region, based on the standard definition of a gene, in which mutations are unconditionally deleterious, are continuously arising, have partially recessive and non-complementing effects on phenotype (analogous to what is widely observed for most Mendelian disorders), and are interspersed with neutral markers that can be genotyped. Genes evolving according to this model exhibit a characteristic GWAS signature consisting of an excess of marginally significant markers. Existing tests for an excess burden of rare alleles in cases have low power while a simple new statistic has high power to identify disease genes evolving under our model. The structure of linkage disequilibrium between causative mutations and significantly associated markers under our model differs fundamentally from that seen when rare causative markers are assumed to be neutral. Rather than tagging single haplotypes bearing a large number of rare causative alleles, we find that significant SNPs in a GWAS tend to tag single causative mutations of small effect relative to other mutations in the same gene. Our results emphasize the importance of evaluating the power to detect associations under models that are genetically and evolutionarily motivated.

## Author Summary

Current GWA studies typically only explain a small fraction of heritable variation in complex traits, resulting in speculation that a large fraction of variation in such traits may be due to rare alleles of large effect (RALE). The most parsimonious evolutionary mechanism that results in an inverse relationship between the frequency and effect size of causative alleles is an equilibrium between newly arising deleterious mutations and selection eliminating those mutations, resulting in an inverse relation between effect size and average frequency. This assumption is not built into many current models of RALE and, as a result, power calculations may be misleading. We use forward population genetic simulations to explore the ability of GWAS to detect genes in which unconditionally deleterious, partially recessive mutations arise each generation. Our model is based on the standard definition of a gene as a region within which loss-of-function mutations fail to complement, consistent with the multi-allelic basis for Mendelian disorders. Our model predicts that it may not be uncommon for single genes evolving under our model to contribute upwards of 5% to variation in a complex trait, and that such genes could be routinely detected via modified GWAS approaches.

## Introduction

Genome-wide association studies (GWAS) genotype upwards of 500,000 common SNPs and test for allele frequency differences in case/control panels consisting of several thousand individuals. Such studies have identified highly significant and replicable associations, and as a result have uncovered entirely new pathways contributing to complex disease risk (http://www.genome.gov/gwastudies/). However, these associations explain only a small fraction of the known heritability of risk for the diseases examined [1]. It is well-known that the GWAS paradigm of testing for associations primarily using intermediate frequency markers has high power to identify an association only if disease causing alleles are also at intermediate frequency [2]. This “missing heritability” has led to speculation that a new round of GWAS should be designed to detect rarer variants of presumably larger effect. The potential importance of rare alleles of large effect (RALE) is supported empirically by studies that have carried out deep resequencing of candidate gene exons and observed an excess of rare radical amino acid polymorphisms in cases relative to controls for a variety of diseases (HDL cholesterol levels [3], susceptibility to colorectal adenomas [4], LDL cholesterol levels [5], triglyceride levels [6], folate metabolism [7], and hypertriglyceridemia susceptibility [8]. Collectively, these studies suggest the possibility that the same sort of genetic heterogeneity commonly observed for Mendelian disorders [9], [10]) may characterize complex disease.

A weakness of the RALE model for complex disease variation is that it is not a population-genetic model, but rather an easy to understand verbal model. As a result it does not make quantitative predictions concerning the nature of genetic variation at the genes underlying complex disease, neither in terms of the number of causative alleles, their frequencies and effects, nor in terms of the patterns of linkage disequilibrium (LD) between causative alleles and linked neutral markers. Ideally, the predictions of various RALE models would come from explicit population-genetic models of disease, with concrete assumptions about the fitness effects of causative mutations and the relationship between phenotype and fitness determining the frequency of causative mutations in the population. To date, Prichard's [11] work is the best attempt to model the impact of the equilibrium between mutation and selection on the frequencies of disease-risk mutations. His model generates scenarios where the genetic basis of a complex disease consists of many rare mutations at different sites within a gene, with the frequency of causative mutations being determined by the balance of mutation and natural selection [12]. His model is a case for which the power of standard association tests is greatly reduced [2], [13], [14].

Pritchard's [11] work did not model intragenic recombination, nor track neutral mutations partially-linked to causative deleterious mutations. Thus, Pritchard was unable to explicitly address the power of GWA studies, which genotype both causative and linked neutral SNP markers throughout the genome. This question instead requires the use of explicit simulations of an evolving phenotype and its molecular basis. In recent work, the application of population-genetic principles to understanding the properties of GWAS have been highly heterogeneous. For example, some authors have modeled the frequencies of risk alleles in a region as independent random variables, as opposed to simulating a recombining region [15]–17. In these studies, the genotype/phenotype relationship is based on arbitrary choices as to the number of causative mutations [15]–[17]. A second set of studies have simulated recombining regions using coalescent simulations without selection [e.*g.*, 18] to generate a large set of haplotypes from a model with explicit assumptions about demography (these assumptions vary from study to study) [19]–[21]. The authors then selected an arbitrary number of mutations from arbitrary frequency ranges to be causative mutations, and arbitrary effect sizes are assigned. Finally, some studies have used forward simulation machinery [*e.g.*, 22] to simulate multiple partially-linked deleterious mutations subject to natural selection in a region interspersed with neutral mutations [23]–[25]. In these forward simulations, fitness values were assigned to particular sites according to genotype, and the final fitness of a diploid is typically either the sum or product of fitness effects over deleterious mutations. The simulation output is then used to map genotype to phenotype using either an arbitrary model 24,25 or an explicit quantitative genetic model [23]. Although this last class of models represents the most sophisticated application of evolutionary simulations, they are still limited in that the phenotype itself is not the target of natural selection (as is the case in [11]), and thus the simulated distributions of phenotypes are not the outcome of an evolutionary process (even though the underlying mutation frequencies are).

Here, we propose an explicit model of a quantitative trait subject to natural selection, with case/control status treated as a liability trait. Our model is similar to that of Pritchard [11] in that the frequencies of deleterious mutations are the result of the balance between mutation and natural selection [12], and similar to other recent work [23], [24], [25] in employing explicit forward simulations. We depart from existing work using forward simulations in two important ways. First, the phenotype itself determines fitness and thus is the target of natural selection. Second, our model of gene action is based on the standard definition of a gene as a region in which recessive mutations fail to complement [26], such that affected individuals will generally be a trans-heterozygote for causative mutations (*e.g.* heterozygous for at least two different causative mutations at different positions in the gene region), as is commonly-observed for Mendelian disorders [10], [27]. Our model of fitness is, therefore, based on the partial recessivity of haplotypes and not on the standard population-genetic assumptions of multiplicative or additive fitness across individual mutations. Under these standard assumptions (used in simulation programs such as [22]), fully-recessive mutations at different positions complement one another in terms of fitness (e.g., the fitness of a trans-heterozygote is the same as a diploid that is homozygous for wild-type alleles at both sites) and, therefore, the different deleterious mutations within a simulated region are, themselves, different genes (*sensu* [26]). We develop a novel forward simulation and use it to simulate a “typical” 100 kilobase region of the human genome, tracking both causative deleterious and non-causative neutral mutations. In our simulations, disease risk is due to an underlying continuously varying liability score, with a causative disease “gene” contributing ∼5% to variation in that score. Given the size of the region considered, the idea that the vast majority of mutations are neutral, and the nature of gene action being modeled, our models are most consistent with mutations impacting both the structural product and the *cis*-regulatory regions controlling expression of a gene contributing to risk of a complex disease. We use our simulation machinery to explore the population-genetic signals of selection against causative sites, and to explore the power of GWAS to test the hypothesis that variation within a gene region contributes to complex disease.

Our model results in gene regions evolving under the “allelic heterogeneity” model involving many non-complementing risk mutations segregating within a gene region. Since the 1990s, many human geneticists believed that this model was likely to explain complex variation [9], [ pg 492; 10]. Under this model, complex traits are genetically analogous to Mendelian disease genes, but the mutations are simply less penetrant due to other genes and environmental variation impacting the trait [9, Chapt. 14&15]. Our model results in weak selection against causal variants, with no detectable average effect on patterns of variation at linked neutral sites. A major finding is that statistical tests designed to detect an overall greater number of rare alleles in cases have very low power (such tests have been argued to have high power to detect RALE), and less power than the standard single-marker logistic regression assuming an additive model. The frequencies of significant associations from single-marker tests applied to our simulated GWAS involving common markers are consistent with empirical observations from real GWA studies [28], in contrast to previous results based on simulating RALE as neutral [19], [28]. We propose a simple statistic based on the excess of marginally significant markers in a region, and find that it has higher power to detect associations than other tests considered, although the power of the SKAT package [21], [29], [30] can have comparable power. Finally, in our simulations, the explanation for missing heritability in current GWAS in that significantly associated common markers tend to be associated with causative mutations with relatively small effect sizes, but fail to tag rarer variants of larger effect. The observation that more subtle-effect variants can drift to higher allele frequencies is consistent with population-genetic predictions of an inverse relationship between average frequency and effect on fitness [*e.g.*, 23], which we expect to be a general property of any model involving mutation-selection balance. This explanation for missing heritability differs from the hypothesis of “synthetic associations” arising when RALE are assumed to be neutral [19]. The simulated datasets represent an important resource for evaluating the power of novel test statistics under the heterogeneity model.

## Results

### An intuitive gene-based model results in weak selection against affected individuals

**Figure 1:**
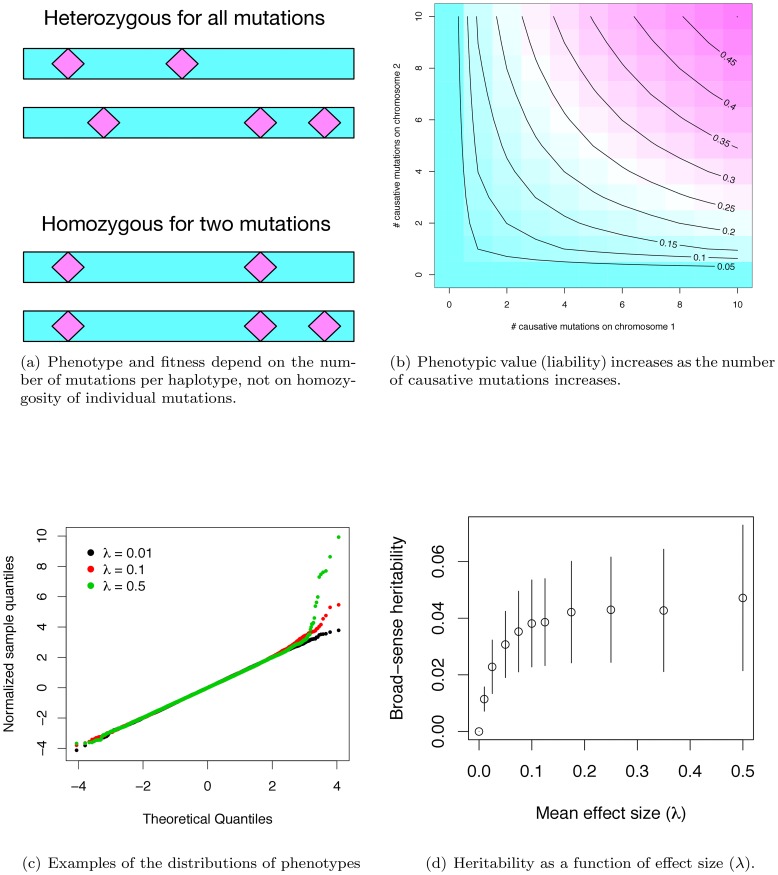
Phenotypes under the gene-based model. (a) Phenotype depends only on the number of causative mutations present on each haplotype, and not on whether an individual is homo- or heterozygous for particular mutations. Thus, the two diploids shown are equivalent in their expected phenotype, as both diploids contain one haplotype with two causative mutations, and a second haplotype with three such mutations. (b) Phenotype is calculated as the geometric mean of the effects of each haplotype, and is therefore determined primarily by the haplotype closest to wild-type. The panel shows the expected phenotype for diploids with different combinations of mutations on each haplotype, assuming a constant effect size of 0.05 per mutation. (c) Quantile-quantile plots of phenotypes resulting from the simulation. The x-axis is the quantiles of a unit Gaussian, and the y-axis is the z-score normalized quantiles observed in a simulated population. For three different parameter values, the phenotypes of 20,000 diploids from a single simulated population are shown. At moderate average effect sizes (λ) (0.10 in the panel), there tends to be an excess of individuals with modestly-large phenotypes, whereas with large λ, a population typically contains proportionally more individuals with large phenotypic values. (d). Broad-sense heritability as a function of λ, the mean effect size of a causative disease mutation. Plotted are the mean values ±1 standard deviation, calculated from the simulation output.

Under our gene-based model, the effect size of a causative mutation is exponentially-distributed with mean λ (λ = 0 implies a mutation that does not contribute to a complex disease phenotype), and the effect of a maternal or paternal haplotype is additive over causative mutations. The phenotype of a diploid is the geometric mean effect of the maternal and paternal haplotype plus a random Gaussian environmental effect scaled so that the gene-region being modeled accounts for some fraction of the total disease burden. Figure 1a shows the difference between our model of gene action (non-complementation of loss-of-function mutations) and the standard population-genetic assumption that mutation effects are multiplicative. By setting the contribution of a gene to an individual's phenotype equal to the geometric mean of the maternal and paternal haplotypes, the haplotype closer to being mutation-free dominates the genotypic effect (Figure 1b), resulting in partially recessive model of gene-action (Figure S1a), which is empirically supported for mutations of moderate effect [31]–[34]. As in Pritchard's work [11], the distribution of effect sizes at causative sites at equilibrium is not equivalent to the distribution of newly arising mutations. Rather, the frequency distribution of causative mutations at equilibrium is determined by a balance of mutation, genetic drift, and natural selection. This contrasts with other attempts to model the frequency distribution of causative mutations using an arbitrarily defined statistical distribution (as in [17]) or arbitrary numbers of causative mutations [19], [21], [29]. Our approach also differs from previous approaches in that the number of causative mutations in a region is a random variable, as opposed to a fixed an arbitrary quantity [15], [16], [19], [21], [29], [35]. Insomuch as the assumptions of our model are correct, we are properly specifying the equilibrium distribution of the number, frequencies, and effects of causative mutations, as well as the extent of LD between causative and linked neutral sites.

In our simulated populations, liabilities are close to normally distributed, except in the extreme “diseased” tail, where there is a slight excess of extremely affected individuals (Figure 1c). Further, the fitness of an affected individual is generally high (Figure S1b), meaning that even individuals with the most extreme liabilities are capable of approximately normal reproduction. Although there is considerable uncertainty surrounding the distribution of fitness in human populations, and the strength of purifying selection on complex diseases remains a subject of debate, our model is consistent with the idea that purifying selection on complex disease phenotypes is generally weak, as has been claimed in the literature [36].

### The heritability due to single genes under our model

Given the computational demands of forward simulation, we focus our attention on a set of parameters (see Methods) that results in the proportion of total phenotypic variation in the population attributable to the focal gene region reaching a plateau at ∼4% as λ increases to ∼0.075–0.10 (Figure 1d). Although our simulations assume a uniform rate of crossing over per generation, heritability similarly plateaus at ∼4% for a region with zero recombination (Figure S1c) when using the same mutational parameters as in Figure 1d. Since the power to detect an association depends on the recombination rate between genotyped markers and causative mutations, we present results only for the case of no recombination (representing extreme “cold” regions of recombination) and for a region recombining at a uniform rate representing the genome average. Thus, while we are not explicitly modeling hotspots of recombination, we view the results as broadly-applicable on average.

The value of heritability at the plateau depends on the model parameters. Plateau height is approximately linear as a function of the deleterious mutation rate (Figure S2a), holding all other parameters the same as Figure 1c. Thus, holding the per-site neutral mutation rate constant, the heritability due to a gene region is a function of the proportion of sites mutable to disease alleles and the physical size of a gene. Further, we can “tune” the expected value of the heritability at the plateau by increasing or decreasing the value of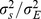 (the ratio of the variance in fitness and variance due to random effects) in a manner broadly consistent with the house-of-cards model for the maintenance of quantitative genetic variation under mutation selection balance ([37]; Figure S2b and S2c). Thus, in spite of the considerable uncertainty in the values of μ_d_ (the deleterious mutation rate, or the product of proportion of sites mutable to a causative allele and the size of a gene for a constant per site mutation rate) an λ (the average effect size of newly arising exponentially distributed deleterious mutations), our model is able to generate a single gene of small to large effect contributing to disease risk for plausible parameters (Figure S2a). It is additionally noteworthy that the stochastic variation around the expected gene-specific heritability (conditional on μ_d_ and λ) is quite large. This implies that even for parameter combinations that predict equilibrium heritability values of ∼2% (*e.g.* Figure S2a), 2–9% of genes sharing these parameters will *each* account for >5% of the total phenotypic variation in a complex trait (data not shown). Therefore, despite our focus on parameter values that result in a heritability plateau of ∼4%, parameter values predicting lower plateaus are clearly of interest.

### Population genetic signatures

We examined the frequencies of mutations in a sample of 100 diploids drawn from each of the simulated regions. On average, causative mutations are more rare than expected in the absence of natural selection (Figure S3a–S3d). The strength of the skew towards rare alleles is stronger with increasing λ, consistent with the observation that at the heritability plateau, larger λ are associated with variation being due to fewer (Figure S3e), more rare (Figure S3f), and larger-effect mutations compared to small λ. In contrast, the site frequency spectrum at non-causative variants is indistinguishable from neutrality irrespective of λ. As the vast majority of polymorphisms are non-causative under our model (Figure S3f), it is unlikely that population-genetic methods would identify these gene regions as abnormal, despite their strong contribution to disease. Thus, under our model there is only a very slight excess of rare alleles observed in case versus controls (Figure S3e), and this is not likely a fruitful signal to look for. It is important to note that this result may not extend to “exon sequencing” where there may indeed be an excess of rare non-synonymous (NS) SNPs in cases relative to controls. If disease is primarily due to rare NS mutations in genes making strong contributions to total risk, the fraction of NS SNPs mutable to causative alleles is likely much greater than the same fraction for a gene region in general (including non-coding regulatory regions) as modeled here.

### Some properties of GWAS signals

**Figure 2:**
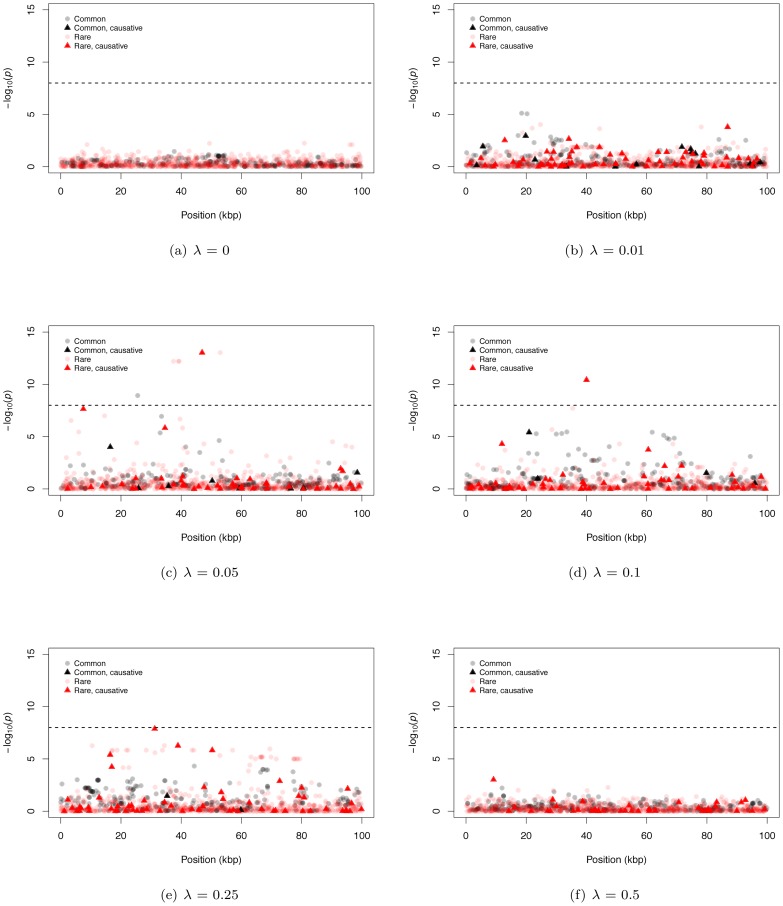
Representative Manhattan plots. The dashed horizontal line corresponds to a p-value of 10^−8^. (a–f) The −log_10_ of the p-value of the logistic regression is shown for representative examples for different mean effect sizes of causative mutations (λ). The plots are separated into four classes of mutations: common neutral and causative variants, which could be typed in a GWAS, and rare neutral and causative variants which would only be directly typed by resequencing.

Figure 2 shows representative Manhattan plots (based on a marker-by-marker logistic regression of 3000 case/control samples) for 100 kb gene regions and different λ (λ = 0 is a no deleterious mutation control simulation). Since we track every SNP in a region, we can distinguish neutral from causative markers as well as common from rare markers. For both large and small λ, it is difficult for markers from either SNP-chips or complete resequencing studies to reach a genome-wide statistical significance threshold (Figure 2a, 2b, and 2f; we assume SNP-chips type exclusively SNPs with a MAF ≥5% and all such SNPs are genotyped). For intermediate λ it is possible for markers to be significant at a genome-wide threshold, and rare markers are more likely to approach significance than intermediate-frequency markers (Figure 2c–2e). For intermediate λ, only a small percentage of rare sites are causative (<10% on average for all λ), yet causative sites are much more likely to be among those markers reaching significance than non-causative sites (17.6% to 31.3% of significant rare markers are causative; Figure S4a). Thus, unlike current GWAS using intermediate frequency markers, under our gene-based model significant associations between rare markers and disease status are likely to identify truly causative mutations.

We also observe examples of significant, common, non-causative markers (e.g. Figure 2c), consistent with current GWAS hits occasionally uncovering genes evolving under our gene-based model. In general, significant common markers are only in strong linkage disequilibrium with a single causative SNP (Figure S4b); similarly, when several common non-causative markers are significant in a single region, they tend to “tag” different causative SNPs (Figure S4c), which themselves tend to be surprisingly common with small effect sizes (Figure S4d). Thus, an individual significant common neutral marker is typically associated with a single causative site of weak effect that has drifted to an intermediate frequency (*i.e.*, an evolutionary outlier). This observed relationship between common SNPs significant in a GWAS and causative alleles is inconsistent with the claim of recently published work that introduced the idea of a synthetic association resulting from a common marker tagging a haplotype(s) harboring an excess of rare causative mutations [19], [38]. It is likely this difference stems from the fact that the synthetic association papers assume causative alleles are neutrally evolving, yet narrowly constrained in allele frequencies, whereas here we assume causative alleles are deleterious with equilibrium frequencies and patterns LD ultimately determined by evolutionary forces.

### The power of marker-by-marker GWAS to identify genes

**Figure 3:**
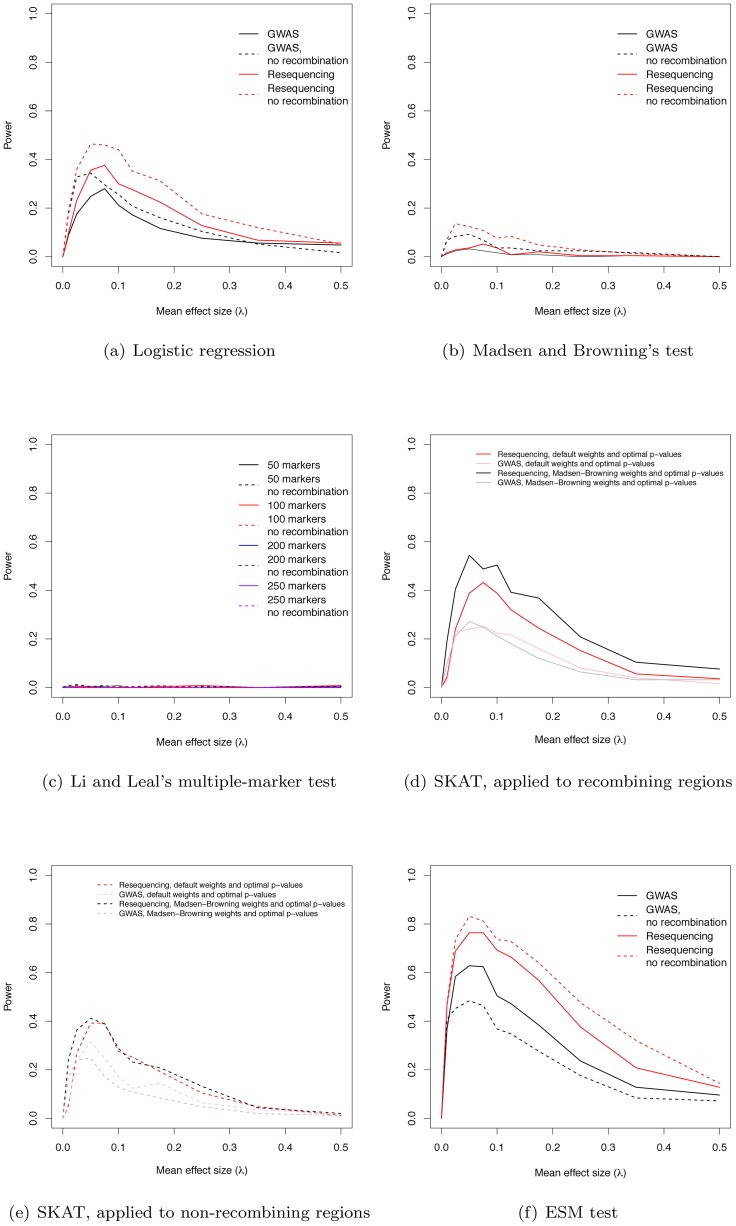
Power to identify regions containing causative mutations. (a) The power of the logistic regression in GWAS and resequencing studies at significance threshold α = 10^−8^. (b) The power of Madsen and Browning's [16] test (at α = 10^−6^). (c) The power of Li and Leal's [15] multiple-marker test (at α = 10−6). (d) Power (at α = 10−6) using the SKAT software package, applied to data from recombining regions. (e) Power (at α = 10^−6^) using the SKAT software package, applied to data from non-recombining regions. (f) The power of the ESM test (at α = 10^−6^, see Methods) in GWAS and resequencing studies.

We estimated the power of the widely-used logistic regression approach to identify regions containing at least one significant marker. For the parameters simulated, power maximizes at 28% in a GWAS using common markers and at 38% in a resequencing study, when λ = 0.075 (Figure 3a). When λ = 0 (no deleterious risk mutations present), power is 0 at significance level α = 10^−8^. Further, the cumulative distribution of p-values for λ = 0 is a line with a slope less than one, indicating that the logistic regression test is conservative when applied to our simulated data (data not shown). For small values of λ>0, broad-sense heritability is also lower (Figure 1d), resulting in less power. As λ increases broad-sense heritability reaches a plateau (Figure 1d), but after that plateau is reached power begins to decrease as causative variants become more and more rare in the general population (Figure 3a, Figure S4). Thus, depending on the value of the largely unknown parameter λ, current approaches based on common markers have limited power to identify genes harboring causative deleterious alleles, consistent with the idea that some of the “missing heritability” associated with current generation GWAS is due to RALE. Although we are not the first to point this out (*c.f.* [2]), complete resequencing of cases and controls may only yield a modest improvement in power under a marker-by-marker GWAS (Figure 3a). Finally, the power of GWAS and resequencing studies to identify gene regions is only slightly higher in a region of zero recombination (Figure 3a).

### The power of existing tests for RALE

We applied Madsen and Browning's rank-sum test [16], Li and Leal's multiple marker test [15], and the software package SKAT [21], [29], [30] to our simulated data. The first two tests have been proposed to detect an excess of rare alleles amongst cases for gene regions (typically genes, or a fixed physical sliding window). We employed a *p*-value threshold of 10^−6^ (compared to the more conservative 10^−8^ for a SNP-by-SNP GWAS) for these gene-based tests, as they integrate over markers, and thus fewer tests are carried out when doing a genome-wide scan. The Madsen and Browning test results in an excess of small *p*-values (compared to the same test in λ = 0 controls) across a wide range of λ, with the excess being greater in resequencing studies than chip-based GWAS (Figure S5a–S5j), but the *p*-values are rarely small enough to reach genome-wide significance. As a result power maximizes at 5.2% for resequencing studies and intermediate λ (Figure 3b). In contrast, the Li and Leal multiple marker test shows no enrichment for small *p*-values (Figure S6a–S6j), and power <1% for all λ (Figure 3c). In the absence of recombination, the Madsen and Browning test shows a greater excess of small *p*-values and power maximizes at 13.6% when λ = 0.025 (Figure 3b, Figure S7). The power of the Li and Leal test was unchanged in the absence of recombination (Figure S8 and Figure 3c). Both the Madsen and Browning and Li and Leal tests are designed to detect an excess of rare alleles in cases versus controls. However, under our model there is only a very slight excess of rare variants in cases relative to controls at disease genes. This is because the proportion of rare variants that are disease-causing (as opposed to neutral) in a gene region at equilibrium is small and the sampling variance on this proportion is large under the mutation-selection balance model we consider. Although the test statistics proposed by Madsen and Browning and by Li and Leal are reasonable, the information they are exploiting, which depends on a net excess of rare alleles in cases, is generally unable to distinguish cases from controls when applied to our simulated data. We note that it is possible that the Madsen and Browing and the Li and Leal tests would be more powerful when applied to a subset or markers chosen *a-priori* to be potentially functional. However, the high variance in the relationship between effect size and average allele frequency of a causative deleterious marker (see Figure 2 of [23] for the case of multiplicative fitness effects) suggests that the signal-to-noise ratio may still remain low.

The power of the SKAT software to detect associations in recombining regions is shown in Figure 3d. For GWA studies, power peaks at 27.2% when λ = 0.05. The two weighting schemes applied to individual markers (see Methods) result in similar power profiles. For resequencing studies, power maximizes at 54.5% when λ = 0.05, with the power being greatest when using Madsen and Browning's [16] weighting scheme for individual markers (Figure 3d; Madsen Browning weights are not equivalent to the test proposed in [16]). When applied to non-recombining regions, we observe approximately 10% less power across all effect sizes, and the different weighting schemes give similar power profiles for both GWAS and resequencing studies (Figure 3e).

### Our model suggests new alternative statistical tests

An interesting feature of the Manhattan plots (Figure 2) is that for all but the highest values of λ we observe a “genetic signature” of a gene contributing to a complex phenotype that consists of a large number of markers with suggestive, but not globally significant, *p*-values (*e.g.*, contrast Figure 2a with b–e). Upon further examination the majority of the tagged causative mutations are rare in the population and, since they tend to occur on different genetic backgrounds, are only weakly correlated with one-another. This observation suggests that the design and implementation of a statistical test that integrates over approximately independent rare markers located in a gene-region is a fruitful avenue for future research. We applied a new statistical test (ESM, described in Methods) to our simulated gene regions to determine if there is information not currently being exploited by published statistical tests. The ESM test statistic is the sum of the difference in the observed and expected p-values (on a log_10_ scale) of the M most significant markers in a genomic region (see Methods for details). Control simulations with no causative mutations show that a permutation procedure (see Methods) results in the correct distribution of *p*-values (Figure S10a) and a power of zero at a significance threshold of 10^−6^ (Figure 3f). The ESM statistic is the most powerful of all the statistics evaluated under either a common marker GWAS or resequencing experimental paradigm, with complete resequencing giving the highest power than GWAS over all values of λ. When only common markers are genotyped the marker-based logistic regression and SKAT are the second and third most-powerful approaches respectively (Figure 3a and 3d), whereas SKAT is the second most-powerful approach under resequencing (compare Figure 3d to Figure 3f). For λ in the range of 0.05–0.15, the power of the ESM test can approach 77% and the power only drops below 20% for λ>0.35. In the absence of recombination, power can be as high as 82% for intermediate λ (Figure 3f). The statistical properties of this statistic are detailed in Figures S9 and S10. Such a test could be implemented genome-wide using a sliding-window or a gene-centric approach. We developed this test to serve as an illustrative example of a test that attempts to integrate information over a gene-region, and we suspect that more sophisticated tests could be designed to detect the cumulative effects of rare variants in a gene region.

### The “missing heritability problem” of current GWAS

Goldstein and colleagues [19], [38] have proposed that common variants may be tagging haplotype(s) harboring several low frequency causative alleles. However, their model assumes that causative mutations may be modeled by placing them on neutral genealogies within a small window of frequencies [19]. When both the effect sizes and allele frequencies of causative polymorphisms are random outcomes of the evolutionary process, we observe that significant common variants tend to tag a single causative variant (Figure S4c) of small effect that has drifted to modest frequency (Figure S4d). If RALE are deleterious instead of neutral, this observation casts doubt on the claim that common variants generally tag haplotypes harboring several low-frequency causative alleles. Our results suggest a different interpretation of missing heritability, one which is consistent with standard population-genetic predictions of an inverse relationship between frequency and the effect size of a deleterious mutation [11], [12], [23]. Conditional on observing a significant common marker near a gene experiencing recurrent deleterious mutations, that marker likely tags a single causative SNP whose effect size is small enough (and therefore selection weak enough) that that mutation drifted to high frequency. Thus, the missing heritability in our simulations is due to that single association tagging only one out of several causative variants segregating in a region, with the effect size of that tagged mutant being smaller than that of others segregating in the region. We note that this phenomenon is not unique to the model simulated here. Any evolutionary model with a distribution of negative selection coefficients associated with newly arising deleterious mutations will predict an inverse relationship between population frequency and selection coefficient, conditional on a variant segregating in the population (e.g. [23]). Consistent with this hypothesis, the mean number of singletons on a haplotype defined either by the number of copies of the derived allele at the most significant marker in a GWAS, or by the number of copies of the derived allele at a SNP not associated with case control status, do not differ appreciably for the parameters considered here (Figure S11). Therefore, under the gene-based mutation-selection balance model considered here, significant associations are not tagging haplotypes with unusual numbers of rare alleles on average (c.f. 19).

**Figure 4:**
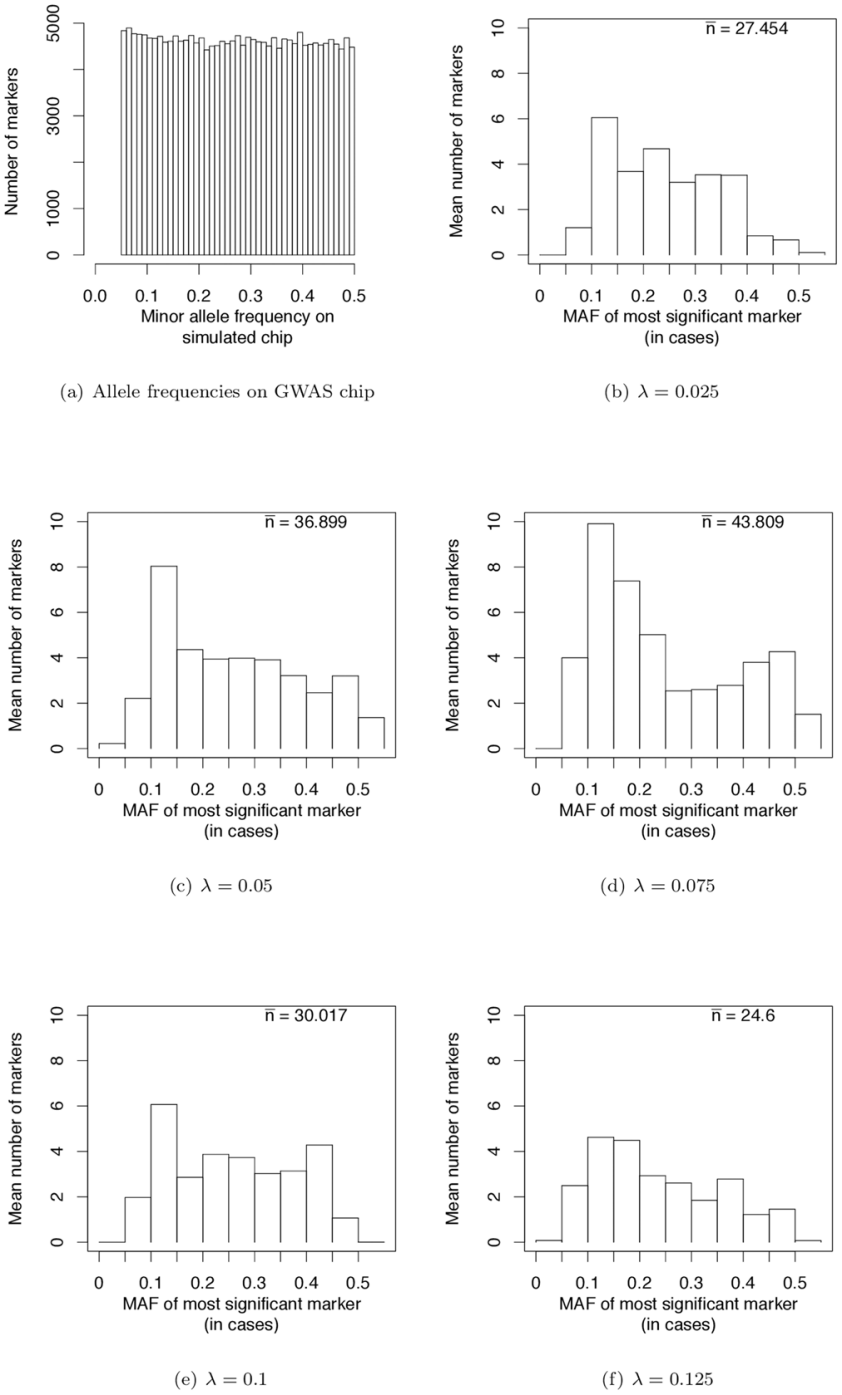
Frequencies of most significant markers (based on the logistic regression test) in GWAS based on genotyping panels of previously ascertained SNPs. (a) From our simulated case-control studies, we randomly-sampled markers in order to mimic the ascertainment of common markers typical of current GWAS, which resulted in a uniform distribution of minor allele frequencies. The distribution shown here is summed across all replicate simulations of a gene region. (b–f) Monte Carlo estimates of the expected number of most-associated markers in different frequency intervals for different values of λ (the mean effect size of a causative mutation). The x-axis represent the frequency of the minor allele (defined in the general population) in the cases. In each panel, 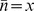 is an estimate of the expected number of replicates (out of a total of 250) containing at least one significant marker using an imperfect SNP chip.

Wray *et al.* [28] have pointed out that the allele frequencies of strongest associations in current GWAS are nearly uniformly distributed (see their Figure 2a). We sampled markers from our simulated case/control samples such that the MAF on GWAS chips are uniformly distributed (Figure 4a). This distribution matches the simulated MAF distribution in [28] and that seen on SNP chips. When we use such a SNP chip to carry out GWAS under our evolutionary model of RALE, the resulting MAF distribution at significantly associated SNPs appears rather uniform, with a slight excess toward intermediate MAF for some λ (Figure 4b–4f). Thus our simulations are consistent with the results of current GWAS, and inconsistent with Dickson and colleagues [19], [38] as represented in [28] (c.f., their Figure 2). We conclude that many currently reported associations presumably reflect bona fide intermediate frequency variants, and that the “missing heritability” problem may arise from GWAS being biased towards detecting associations with causative mutations of small effect relative to the average effect size at a causative gene.

## Discussion

Risch [39], [40] presented an early and influential attempt to model the genetics of complex traits and to frame the model in terms of measurable parameters such as relative risk. For a given locus, he considered the case of a single risk allele (the product of a single mutational event some time in the past) with some specified effect size. Risk alleles at different genes interact multiplicatively to generate an individual's phenotype, a common assumption in multi-locus models in evolutionary biology [41], [42]. Risch and Merikangas [13] used this model to calculate the power to detect a risk allele at an arbitrary frequency in the population. Pritchard [11] was the first to add explicit evolutionary considerations to this model, extending Risch's model to the case of a constant-size, randomly-mating population subject to recurrent mutation to risk alleles at multiple loci (with constant effect sizes of risk alleles at a single locus, but varying across loci), multiplicative interaction between loci, and natural selection against risk alleles. In Pritchard's model, the equilibrium frequency distribution of the risk allele class at a single locus is known from population genetics theory ([43], also see equation 1 of [11]), and the frequency of the risk allele class is the sum of the frequencies of the individual susceptibility alleles that have arisen independently at different positions in a non-recombining region. Pritchard's model was an important conceptual advance, allowing the frequencies of the risk allele class to be the random output of the interplay between recurrent mutation, genetic drift, and natural selection. However, due to computational constraints, Pritchard did not explicitly track the frequency of each individual mutation within the risk allele class, nor did he incorporate neutral markers into the model. These two limitations, and the assumed lack of recombination within loci, prevented him from explicitly evaluating the power to detect associations in the case where risk alleles at a single gene are the result of different mutational events embedded in a genomic region consisting largely of linked neutral SNP markers.

Since Pritchard's [11] paper, the application of population-genetic principles to our understanding of the properties of GWAS has been heterogeneous. Rather than employing explicit simulations of the evolution of a disease phenotype, recent studies have employed a variety of approximations ([11], [15]–[17], [19]–[21], [24]–[25], [29], also see Introduction), largely due to computational constraints, and possibly due to the lack of appropriate simulation machinery (but see [44]). As a result, much of the theoretical/statistical current literature on RALE does not incorporate a notion of a gene (*e.g.* [26]) and statistical methods are rarely tested on simulated data that can be described as outcomes of a biological or evolutionary process. Therefore, to more accurately model the ability of GWAS to identify a gene-region harboring RALE, new evolutionary models of gene action are required that are based on a standard well-accepted definition of a gene [26].

Several studies that have carried out resequencing of candidate gene exons in case/control samples have observed an excess of rare non-synonymous mutations in the cases [3]–[8]. Implemented on a genome-wide scale, this “exomics” approach to the genetic dissection of complex traits would most certainly pay dividends [45]. However, it is important to note that scanning for an excess of rare variants within cases may be less fruitful when variants cannot be classified *a priori* as putatively causative (*e.g.* focusing on amino acid variants in coding regions). Our model indeed suggests that tests focusing on detecting such an excess of rare mutations in cases have low power when there are no *a priori* weights applied to different sites within a region and when causative mutations are a small fraction of the total number of variants in a region (Figure 3b–3c, Figure S3e–S3e). These assumptions are likely to be satisfied if some fraction of complex disease is due to mutations in *cis*-regulatory regions and, thus, intuition gained from scanning for RALE in exons may be misleading.

Our model is consistent with the hypothesis that many rare variants could exist at a relatively small number of genes, and as a class those variants are likely to make a measurable contribution to the variation in complex traits. It is not unreasonable to assume that those variants are partially recessive and partially fail to complement one another when located in the same gene. An important aspect of our model is that causative mutations may be located anywhere in a large gene region that includes regulatory and splicing control regions, and causative mutations are not limited to point mutations. We show that simple extensions to current marker-by-marker tests have considerable power to detect genes harboring such variants. GWAS employing common markers have harvested the “low-hanging fruit” associated with intermediate frequency causative variants. In light of mounting evidence that common variants only explain a small fraction of the genetic variation in complex disease phenotypes, it behooves us to design experiments that have reasonable power to uncover the genetic architecture of complex traits under specific population-genetic models purporting to explain the existence of variation in these traits. Forward simulations that can track entire gene regions under intuitively appealing models of gene action and fitness allow us to assess the power of different experimental designs.

## Materials and Methods

### Forward simulation

We implemented a forward-time simulation of a Wright-Fisher population with mutation following the infinitely-many sites model [46], recombination, and selection occurring each generation. We simulated a population of N = 20,000 diploids with a neutral mutation rate of μ = 0.00125 per gamete per generation, and a recombination rate of r = 0.00125 per diploid per generation. These values correspond to the scaled parameters θ = 4Nμ = 100 and ρ = 4Nr = 100, and thus correspond to a “typical” 100 kilobase region of the human genome. The mutation rate to causative mutations was μ_d_ = 0.1 µ per gamete per generation. In our model, causative mutations are treated as SNPs for simplicity, but should be viewed more generally as genetic events (including copy-number variants and transposable element insertions) that we assume to be detectable via a chip or resequencing assay.

We note that there are a variety of forward-time simulation programs in the literature. However, the majority of these either simulate non-gene-based models [22], [47], [48], models involving only unlinked makers [49], or only neutral models [50]. Further, none of them simulate the explicit genotype-phenotype relationship assumed here (see Introduction).

### Model of disease and fitness

An individual carries c_1_ and c_2_ causative mutations on each haplotype. The effect size of the i^th^ mutation on the j^th^ haplotype is 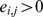, and the phenotype of an individual is P = 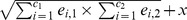 where x is a Gaussian deviate with mean 0 and standard deviation σ_e_, which we fix at 0.075 in the simulations. In words, the phenotypic effect of a single haplotype is additive over causal mutations, and the phenotype of an individual is the geometric mean of the effects of each haplotype plus Gaussian noise. Since phenotypes are continuous they represent the underlying liability of developing a disease [51, chapter 18]. When we refer to heritability and phenotypic distributions in the population in the text, such references are in regards to these liabilities. The phenotypes are under Gaussian stabilizing selection with a standard deviation of σ_s_ = 1, and w, the fitness of a diploid, is proportional to 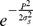.

In our simulations, the effect sizes of causative mutations are exponentially distributed with means of λ = 0, 0.01, 0.025, 0.05, 0.075, 0.1, 0.125, 0.175, 0.25, 0.35, or 0.5. For each λ>0, we performed 250 independent simulations. For an effect size of 0, representing “control” simulations where there is no genetic contribution to risk, we simulated 1000 independent replicates. All simulations were run for 8N generations prior to sampling.

### Exploring the parameter space

For the parameters μ = 0.00125/gamete, μ_d_ = 0.1 µ, σ_s_ = 1, σ_e_ = 0.075, and r = 0.00125/diploid or 0, we simulated both neutral and causative markers, allowing us to examine the properties of GWAS in detail. In order to reduce computational time, for all other parameter values explored, we set μ = 0 (i.e., no neutral mutations were simulated) and only simulated the causative sites. By not simulating the neutral mutations, simulations run orders of magnitude faster, allowing us to look at heritability across a broader parameter space.

### Case-control studies

For each simulated population, 3000 cases and 3000 controls were sampled. A case was defined as being in the upper 15% of the phenotypic distribution, and controls were within 1 standard deviation of the population mean. For each case-control panel, we define a GWAS to include all markers present in the panel with a minor allele frequency ≥5%, and a resequencing study to include all markers. For both types of study, we performed a logistic regression of case/control status onto genotype under an additive model.

The significance threshold used was 10^−8^, representing a typical cutoff used in current GWAS [19], [52]. The power simulations refer to these case/control samples, with power defined as the proportion of replicate simulations with at least one marker in the gene region exceeding the genome-wide significance threshold.

### Data set availability

The forward simulations required approximately six weeks on a cluster of 96 computing cores (AMD Opteron 6168, 1900 Mhz). To facilitate the further development of tests for detecting associations in gene regions, we have made all source code, forward simulation output, and case/control files available online at http://www.molpopgen.org/ThorntonForanLongPLoSGenetics.html.

### Gene/region-based tests of association

In addition to the single-marker test, we also applied several existing and one new test of an association of genotype with case/control status to our simulated data. For tests applied to a set of markers within a genomic region, the significance threshold should be less conservative than the 10^−8^ used for the single-marker test. Our simulated data are 100 kilobase regions, from a genome of approximately 3×10^9^ base pairs, giving 3×10^9^/10^5^ = 3×10^4^ non-overlapping windows. A conservative significance threshold would thus be 0.05/(3×10^4^) = 1.67×10^−6^. Here, we take p< = 10^−6^ as the significance threshold for all region-based tests (following, for example, [21]).

### Excess of Significant Markers (ESM) test statistic

We developed a statistical test that attempts to integrate significance over marginally significant variants in a single gene. Under the gene-based model, genes harboring causative mutations tend to display such a genetic signature, and the ESM statistic is larger when there are more marginally significant mutations in a genomic region. Given a vector of Fisher's exact test p-values (***p***) comparing allele counts in cases and controls for *M* unique markers (i.e., redundant markers collapsed) from a gene region, we define *Y_1_* to be the largest value of negative base ten logarithm of ***p***, *Y_2_* the second largest, etc. Then our test statistic is:
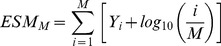
The rationale for the statistic comes from the fact that, if the data were truly drawn from the null model of no contribution of genotype to case/control status, then the expected distribution of p-values is uniform on the interval (0,1]. In other words, for a large number of independent tests applied to data from the null model, the expected fraction of tests with p< = x is x. However, when data truly come from an alternative model, and a test has power greater than the false positive rate, the expected fraction of independent tests with p< = x is greater than x. The ESM statistic is the sum of the difference between the observed and expected *p*-values (on a log_10_ scale) of the M most significant markers in a region.

The test statistic was calculated for two different conditions. First, for GWA studies where, as above, only minor allele frequencies (MAF) ≥0.05 were included. The second condition assumed complete resequencing of individuals and included all markers. For the latter case, and for GWAS assuming a recombining region, we considered values of *M* = 50, since the value of Z_M_ was generally observed to plateau by this point (averaging the statistic over replicates as a function of M). For GWAS in non-recombining regions, we considered values of *M* = 25, as too few simulations had more than 25 unique markers with the requisite MAF to consider larger *M*. For both GWA and resequencing studies, the minimum count of a minor allele had to be 4 in order for a marker to be included in the analysis.

We have also applied several other “region-based” tests designed to detect a contribution of rare alleles to disease risk within a defined genomic region. The first test is Madsen and Browning's [16] rank-sum test. This test ranks individuals using a score that is a function of how many mutations they carry (weighting the contribution of each mutation to the score by its frequency in the control individuals), and the statistic is the sum of ranks in affected individuals. We calculated the test statistic under Madsen and Browning's “general genetic” model, where the score for an individual at a particular marker equals the number of copies of the minor allele carried by the individual (0, 1 or 2). We chose this model because, under the genetic model that we have simulated, it will be rare for affected individuals to be homozygous for a single causative mutation, implying that the per-marker recessive model of Madsen and Browning would not be appropriate. We applied the test to two treatments of the data—GWAS (MAF>5%), and complete resequencing (no MAF filter).

The second test is Li and Leal's [15] multiple marker test, which amounts to calculating Hotelling's T statistic on a matrix of genotype scores (aa = −1, Aa = 0, AA = 1, where a is the minor allele). We applied this test to either the 50, 100, 200, or 250 rarest variants (by minor allele frequency) present in a case-control panel. The Hotelling T statistic was calculated using the “pseudoinverse” function from the “corpcor” library [53] in R [54] for matrix inversion. In practice, the routines used for the matrix inversions required to calculate the test statistic were numerically unstable for larger numbers of markers, resulting in the absence of a p-value for some replicates.

For Madsen and Browning's, and for Li and Leal's, test statistics, we did not first collapse redundant markers. The rationale for not collapsing is that if a “case” contains, for example, two singleton mutations (*e.g.*, present only once in the entire case/control panel), then those two mutations would count more towards case/control differences in the permutation test than they would in the ESM test statistic. Thus, any differences in the power between ESM and the other two statistics should be viewed as conservative.

Finally, we applied the SKAT software [21], [29], [30] (available from http://www.hsph.harvard.edu/xlin/software.html) to all of our simulated data. We applied the software in two different ways. First, we used default weights on individual markers and the optimal p-value approach described in [30]. Second, we applied the marker weights proposed in Madsen and Browning [16] in combination with the optimal p-value approach. Note that the latter is *not* equivalent to Madsen and Browning's rank-sum test [16], but is simply a variant of the SKAT procedure using a different weighting scheme. Because the data are simulated with no complications such as population substructure, sex-specific effects of risk alleles, etc., the only covariate needed for the assessment of significance is the case/control status of individuals.

### Evaluation of statistical significance

For the ESM, Madsen-Browning, and Li and Leal tests, we assessed statistical significance following the permutation procedure outlined in [16]. Case and control labels were permuted 1000 times, resulting in a permutation distribution of the statistic, 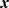. The observed value of the statistic was converted into a z-score (z = 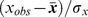), where 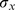 is the standard deviation of the permuted distribution. The distribution of z-scores under the null model of no association with disease is expected to be a unit Gaussian with mean 0 (which we confirmed using control simulations, see panel A of Figures S5, S6, S7, S8, S9, S10), which was used to calculate two-tailed p-values. The SKAT software obtains p-values by fitting a logit model to the data [21] and thus there is no need for permutation.

### Simulating properties of GWAS on imperfect chips

In the analyses described above, we assume that a GWA study is conducted using perfect genotyping technology able to assay 100% of markers with minor allele frequencies >5%. However, the majority of GWAS to date have used genotyping chips that assay a subset of ascertained markers whose minor allele frequencies are uniform in the range of 0.05 to 0.5 [28]. In order to mimic these chips, we resample markers from our case/control panels (described above), including a marker on the “chip” if a uniform random number on the interval (0,1] is < = the heterozygosity of the minor allele in the control population. This sampling results in sample of markers with a uniform distribution of MAF in the desired frequency interval, although some MAF may be >0.5 because the minor allele is defined in the general population, and the control population is a random sample of the general population.

We use these imperfect chips to look at the MAF distribution of the most significant marker (defined by a logistic regression test described above) in a gene region (following [28]). Specifically, we ask what the frequency of the most significant minor allele is in the case population. However, as the number of significant markers per simulated replicate may be quite low (even when using a chip assaying all markers), the resulting distribution of MAF may be very noisy. To reduce this noise, we estimate the expected number of most-associated markers in different frequency bins by randomly sampling 1,000 imperfect chips from each of our 250 replicate case/control populations for each value of λ.

## Supporting Information

Figure S1 Phenotypes under an explicit gene-based model. (a) The model of gene action results in partial recessivity of haplotypes. The panel shows the empirical cumulative distribution of phenotypes that result from our simulations with mean effect size λ = 0.10 per causative mutation (black line), based on 250 independent simulations. Using the output of each simulation, we calculated each individual's phenotype under the standard models used in quantitative genetics–the additive model (red line), recessive model (blue line) and dominant model (purple line) of gene action. These other models were not explicitly simulated. Rather, the haplotype effect sizes output from our gene-based simulation were used to generate phenotypes under these alternative genotype-to-phenotype models. The gene-based model results in a distribution of phenotypes in between that of the additive and recessive models. (b) Average fitness of individuals in the simulations. Red dots show the mean of the population mean fitness. Blue triangles are the average fitness of individuals in the upper 15% of the phenotypic distribution of the population, who were treated as cases in the case-control analyses. The black diamonds are the mean fitness of the least fit individual observed in each simulated population. (c) Mean ±1 standard deviation of broad-sense heritability, as a function of λ. The points with solid lines are the same parameters as in Figure 1d (a region where 4Nμ = 4Nr, where N is the population size, and μ and r the mutation and recombination rates, respectively, and recombination occurs uniformly along the region). Points with dashed lines are from simulations with the same model parameters, but with zero recombination.
(PDF)

Figure S2Broad-sense heritability in different parts of the parameter space. (a) The deleterious mutation rate has an approximately linear effect on broad-sense heritability at large mean effect sizes of causative mutations (λ). All model parameters except the deleterious mutation rate (μ_d_) are the same as in Figure 1d (see Methods). (b) The mean broad-sense heritability was estimated from 250 independent simulated populations for several different parameter combinations, and is shown as a function of λ, the mean effect size of a causative mutation. The open circles are the same data as Figure 1d, and heritability plateaus at approximately 0.04 for large λ. If the magnitude of random effects (σ_e_) is changed (open triangles and solid diamonds), heritability plateaus at different values. However, if 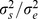, where 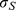 is the variance in fitness, is held constant, heritability plateaus at approximately 0.04 (solid circles and open, upside-down triangles), suggesting that 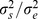 is a critical parameter of the model, as predicted by the house-of-cards model [37]. The magnitude of the heritability at its plateau appears to be linear as a function of μ_d_, plateauing at approximately 0.02 when the deleterious mutation rate is halved. (c) Estimated broad-sense heritability as a function of predicted broad-sense heritability (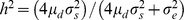) under the house-of-cards model (on a log scale). For 250 replicates with λ = 0.1,0.125, 0.1275, 0.25, and 0.5, the mean heritability was calculated. The median of these five means was used as an estimate of the value of heritability at its plateau (see panel A). Solid circles represent several different parameter combinations where 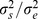
>100, where purifying selection is weak and the house-of-cards assumptions are violated. The solid black line has slope 1 and intercept log(1) = 0. The dashed line is the best-fit line with a slope of 1 and an estimated intercept of −0.6004. This model fits the data better than a model with slope of 1 and intercept of log(1) (p = 2.61×10^−13^,df = 13). Thus, the heritability under the gene-based model is roughly one-half of that predicted under house-of-cards, likely a result of our assumed weak selection [37]. The two open circles are results from simulations where 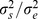
<100, which is in the parameter space covered by house-of-cards, and the observed values are closer, but still less than, the expected values. This difference is likely due to the recessive gene action in the simulations, whereas the house-of-cards model assumes additivity.(PDF)

Figure S3Population-genetic properties of a locus. (a–d) The mean, normalized site frequency spectrum (SFS) of derived mutations is shown for three different mean effect sizes (λ), calculated from a sample of 100 randomly-chosen diploids from each simulated population. Shown are the first ten entries of the SFS for neutral sites (red), causative variants (black), all polymorphisms (dashed blue), and the expected values for a Wright-Fisher population experiencing no natural selection (black circles). (e) Mean (± ¼ standard deviation) of the number of causative mutations per diploid in a case/control panel. For both cases and controls, the mean total number of causative mutations (open circles) and rare causative mutations (diamonds, derived allele frequency <0.05) are shown. (f) Summaries of the amount of variation in the entire population. Here, S_2N_ refers to the mean number of mutations present in the entire population, and 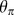 is the average number of differences between two randomly-chosen haplotypes [55]. S_2N_ is plotted on a log_10_ scale. In the absence of selection, the theoretical expectation of S_2N_ is 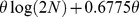 [56, p. 298, equation 9.19]. The excellent agreement between the simulated and the expected value of S_2N_ for neutral markers for all λ shows that the total strength of selection against causative mutations does not result in a loss of variability in the region (because selection is weak on a per-marker basis). For all λ, there is at least a 1 order of magnitude difference in the number of causative and neutral mutations, and 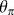 decreases for causative markers as λ increases, indicating that causative mutations are more rare on average as a function of increasing effect size. In the absence of purifying selection, 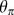 would equal 10 on average at causative sites.(PDF)

Figure S4 Statistical properties of association studies. (a) Average proportion of rare variants which are causative in either the general population, in a case-control panel, or amongst significant markers in a GWAS where individuals are completely sequenced (b) For every neutral, common marker in a GWAS that was significant in a logistic regression test at p≤10^−8^, we measured LD using the r^2^ statistic between the significant marker and all causal markers in the case-control panel, and recorded the top two r^2^ values. The distribution of r^2^ for the top marker is summarized in white boxplots, and the distribution for r^2^ for the second-strongest association is summarized in red. (c) White boxes summarize the distribution of the number of significant, common, neutral markers, conditional on there being at least one such marker. The red boxes summarize the distribution of the number of *unique* causal markers amongst the top r^2^ values for each significant marker. Taken together, panels a and b suggest that significant common markers tend to tag a single causative site. (d) For each of the most strongly-tagged causal mutations making up the red boxes in panel b, the frequency and effect size of each mutant was recorded. The frequencies are summarized in the white boxes, and effect sizes are in red.
(PDF)

Figure S5 Distributions of −log_10_ p-values for the Madsen and Browning [16] statistic. For all panels, the significance threshold of 10^−6^ is shown. (a) The empirical cumulative distribution function (ECDF) of p-values for control simulations with no deleterious alleles. As expected, the ECDF of p-values is a straight line with a slope of approximately 1. (b–k) ECDF of p-values for simulations with non-zero mean effect sizes of causative mutations (λ>0).
(PDF)

Figure S6 Distributions of −log_10_ p-values for the Hotelling T statistic [15]. The statistic was calculated on the rarest 50, 100, 200, or 250 markers. For all panels, the significance threshold of 10^−6^ is shown. (a) The empirical cumulative distribution function (ECDF) of p-values for control simulations with no deleterious alleles. The ECDF of p-values is a straight line with a slope of approximately one when the number of markers is ≥200. (b–k) ECDF of p-values for simulations with non-zero mean effect sizes of causative mutations (λ>0).
(PDF)

Figure S7 Distributions of −log_10_ p-values for the Madsen and Browning [16] statistic with no recombination. For all panels, the significance threshold of 10^−6^ is shown. (a) The empirical cumulative distribution function (ECDF) of p-values for control simulations with no deleterious alleles. As expected, the ECDF of p-values is a straight line with a slope of approximately 1 (b–k) ECDF of p-values for simulations with non-zero mean effect sizes of causative mutations (λ>0).
(PDF)

Figure S8 Distributions of −log_10_ p-values for the Hotelling T statistic [15] with no recombination. The statistic was calculated on the rarest 50, 100, 200, or 250 markers. For all panels, the significance threshold of 10^−6^ is shown. (a) The empirical cumulative distribution function (ECDF) of p-values for control simulations with no deleterious alleles. The ECDF of p-values is a straight line with a slope of approximately 1 when the number of markers is ≥200. (b–k) ECDF of p-values for simulations with non-zero mean effect sizes of causative mutations (λ>0).
(PDF)

Figure S9 Distributions of −log_10_ p-values for the ESM statistic (see Methods). For all panels, the significance threshold of 10^−6^ is shown. (a) The empirical cumulative distribution function (ECDF) of p-values for control simulations with no deleterious alleles. As expected, the ECDF of p-values is a straight line with a slope of approximately 1 (b–k) ECDF of p-values for simulations with non-zero mean effect sizes of causative mutations (λ>0).
(PDF)

Figure S10 Distributions of −log_10_ p-values for the ESM statistic (see Methods) with no recombination. For all panels, the significance threshold of 10^−6^ is shown. (a) The empirical cumulative distribution function (ECDF) of p-values for control simulations with no deleterious alleles. As expected, the ECDF of p-values is a straight line with a slope of approximately 1. (b–k) ECDF of p-values for simulations with non-zero mean effect sizes of causative mutations (λ>0).
(PDF)

Figure S11 A comparison of the burden of risk mutations between significant and non-significant markers in a GWAS. Shown are mean and standard errors of the number of causative singletons in individuals with genotypes defined as having either zero, one, or two copies of the derived allele at the marker most significantly associated (e.g., the smallest p-value) with case/control status in a GWAS analyzed by a single-marker test (red). Pale blue lines show the mean and standard errors of the number of causative singletons associated with zero, one, or two copies of the derived mutation of a marker that is both not associated with case/control status in a logistic regression analysis (p>10^−4^) and frequency-matched to the most-associated marker in the same replicate. Each panel of the figure is labeled by the mean effect size of a causative mutation (λ).
(PDF)
