# Supplementary material for: Properties and Modeling of GWAS when Complex Disease Risk Is Due to Non-Complementing, Deleterious Mutations in Genes of Large Effect
Source: PLoS Genet. 2013 Feb 21;9(2):e1003258. doi: 10.1371/journal.pgen.1003258 (PMC3578756; doi:10.1371/journal.pgen.1003258)
Supplement: Figure S1 — Phenotypes under an explicit gene-based model. (a) The model of gene action results in partial recessivity of haplotypes. The panel shows the empirical cumulative distribution of phenotypes that result from our simulations with mean effect size λ = 0.10 per causative mutation (black line), based on 250 independent simulations. Using the output of each simulation, we calculated each individual's phenotype under the standard models used in quantitative genetics–the additive model (red line), recessive model (blue line) and dominant model (purple line) of gene action. These other models were not explicitly simulated. Rather, the haplotype effect sizes output from our gene-based simulation were used to generate phenotypes under these alternative genotype-to-phenotype models. The gene-based model results in a distribution of phenotypes in between that of the additive and recessive models. (b) Average fitness of individuals in the simulations. Red dots show the mean of the population mean fitness. Blue triangles are the average fitness of individuals in the upper 15% of the phenotypic distribution of the population, who were treated as cases in the case-control analyses. The black diamonds are the mean fitness of the least fit individual observed in each simulated population. (c) Mean ±1 standard deviation of broad-sense heritability, as a function of λ. The points with solid lines are the same parameters as in Figure 1d (a region where 4Nμ = 4Nr, where N is the population size, and μ and r the mutation and recombination rates, respectively, and recombination occurs uniformly along the region). Points with dashed lines are from simulations with the same model parameters, but with zero recombination. (PDF) [file pgen.1003258.s001.pdf]

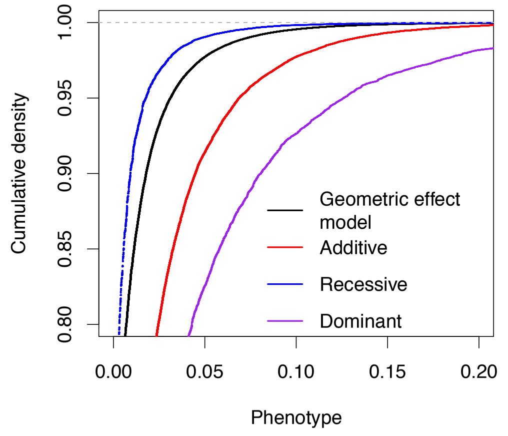

(a) The phenotypic effects of haplotypes are partially recessive.

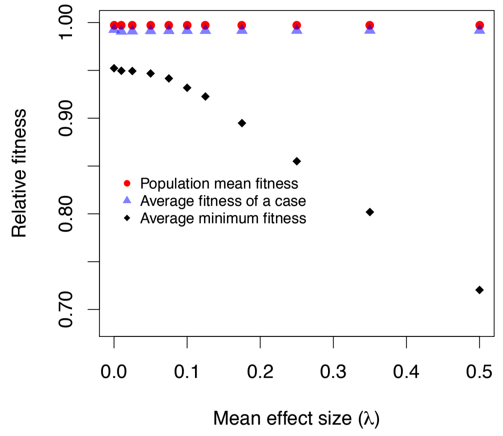

(b) Fitnesses of individuals

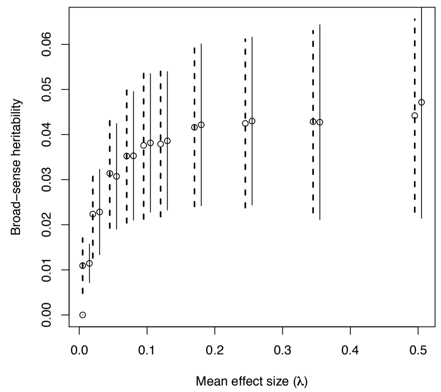

(c) Effect of recombination on heritability

Figure S1: Phenotypes under an explicit gene-based model.
