# Supplementary material for: Properties and Modeling of GWAS when Complex Disease Risk Is Due to Non-Complementing, Deleterious Mutations in Genes of Large Effect
Source: PLoS Genet. 2013 Feb 21;9(2):e1003258. doi: 10.1371/journal.pgen.1003258 (PMC3578756; doi:10.1371/journal.pgen.1003258)
Supplement: Figure S2 — Broad-sense heritability in different parts of the parameter space. (a) The deleterious mutation rate has an approximately linear effect on broad-sense heritability at large mean effect sizes of causative mutations (λ). All model parameters except the deleterious mutation rate (μd) are the same as in Figure 1d (see Methods). (b) The mean broad-sense heritability was estimated from 250 independent simulated populations for several different parameter combinations, and is shown as a function of λ, the mean effect size of a causative mutation. The open circles are the same data as Figure 1d, and heritability plateaus at approximately 0.04 for large λ. If the magnitude of random effects (σe) is changed (open triangles and solid diamonds), heritability plateaus at different values. However, if , where is the variance in fitness, is held constant, heritability plateaus at approximately 0.04 (solid circles and open, upside-down triangles), suggesting that is a critical parameter of the model, as predicted by the house-of-cards model [37]. The magnitude of the heritability at its plateau appears to be linear as a function of μd, plateauing at approximately 0.02 when the deleterious mutation rate is halved. (c) Estimated broad-sense heritability as a function of predicted broad-sense heritability () under the house-of-cards model (on a log scale). For 250 replicates with λ = 0.1,0.125, 0.1275, 0.25, and 0.5, the mean heritability was calculated. The median of these five means was used as an estimate of the value of heritability at its plateau (see panel A). Solid circles represent several different parameter combinations where >100, where purifying selection is weak and the house-of-cards assumptions are violated. The solid black line has slope 1 and intercept log(1) = 0. The dashed line is the best-fit line with a slope of 1 and an estimated intercept of −0.6004. This model fits the data better than a model with slope of 1 and intercept of log(1) (p = 2.61×10^−13^,df = 13). T [file pgen.1003258.s002.pdf]

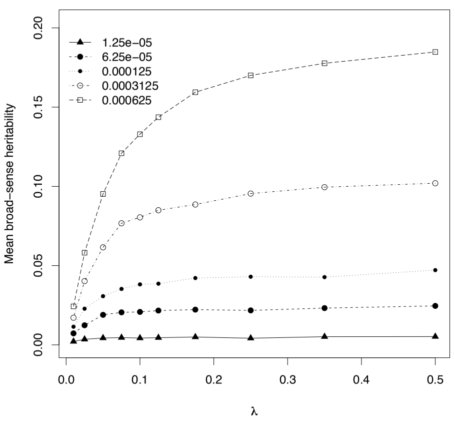

(a) Effect of mutation rate

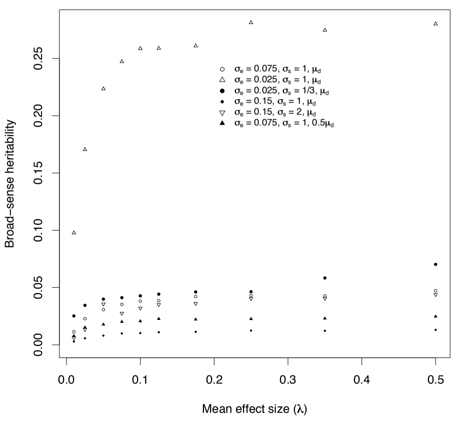

(b) Sensitivity to model parameters

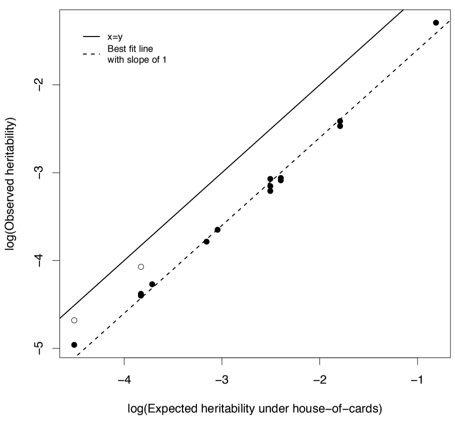

(c) Comparison to House-of-Cards predictions

Figure S2: Broad-sense heritability in different parts of the parameter space.
