# Supplementary material for: Properties and Modeling of GWAS when Complex Disease Risk Is Due to Non-Complementing, Deleterious Mutations in Genes of Large Effect
Source: PLoS Genet. 2013 Feb 21;9(2):e1003258. doi: 10.1371/journal.pgen.1003258 (PMC3578756; doi:10.1371/journal.pgen.1003258)
Supplement: Figure S3 — Population-genetic properties of a locus. (a–d) The mean, normalized site frequency spectrum (SFS) of derived mutations is shown for three different mean effect sizes (λ), calculated from a sample of 100 randomly-chosen diploids from each simulated population. Shown are the first ten entries of the SFS for neutral sites (red), causative variants (black), all polymorphisms (dashed blue), and the expected values for a Wright-Fisher population experiencing no natural selection (black circles). (e) Mean (± ¼ standard deviation) of the number of causative mutations per diploid in a case/control panel. For both cases and controls, the mean total number of causative mutations (open circles) and rare causative mutations (diamonds, derived allele frequency <0.05) are shown. (f) Summaries of the amount of variation in the entire population. Here, S2N refers to the mean number of mutations present in the entire population, and is the average number of differences between two randomly-chosen haplotypes [55]. S2N is plotted on a log10 scale. In the absence of selection, the theoretical expectation of S2N is [56, p. 298, equation 9.19]. The excellent agreement between the simulated and the expected value of S2N for neutral markers for all λ shows that the total strength of selection against causative mutations does not result in a loss of variability in the region (because selection is weak on a per-marker basis). For all λ, there is at least a 1 order of magnitude difference in the number of causative and neutral mutations, and decreases for causative markers as λ increases, indicating that causative mutations are more rare on average as a function of increasing effect size. In the absence of purifying selection, would equal 10 on average at causative sites. (PDF) [file pgen.1003258.s003.pdf]

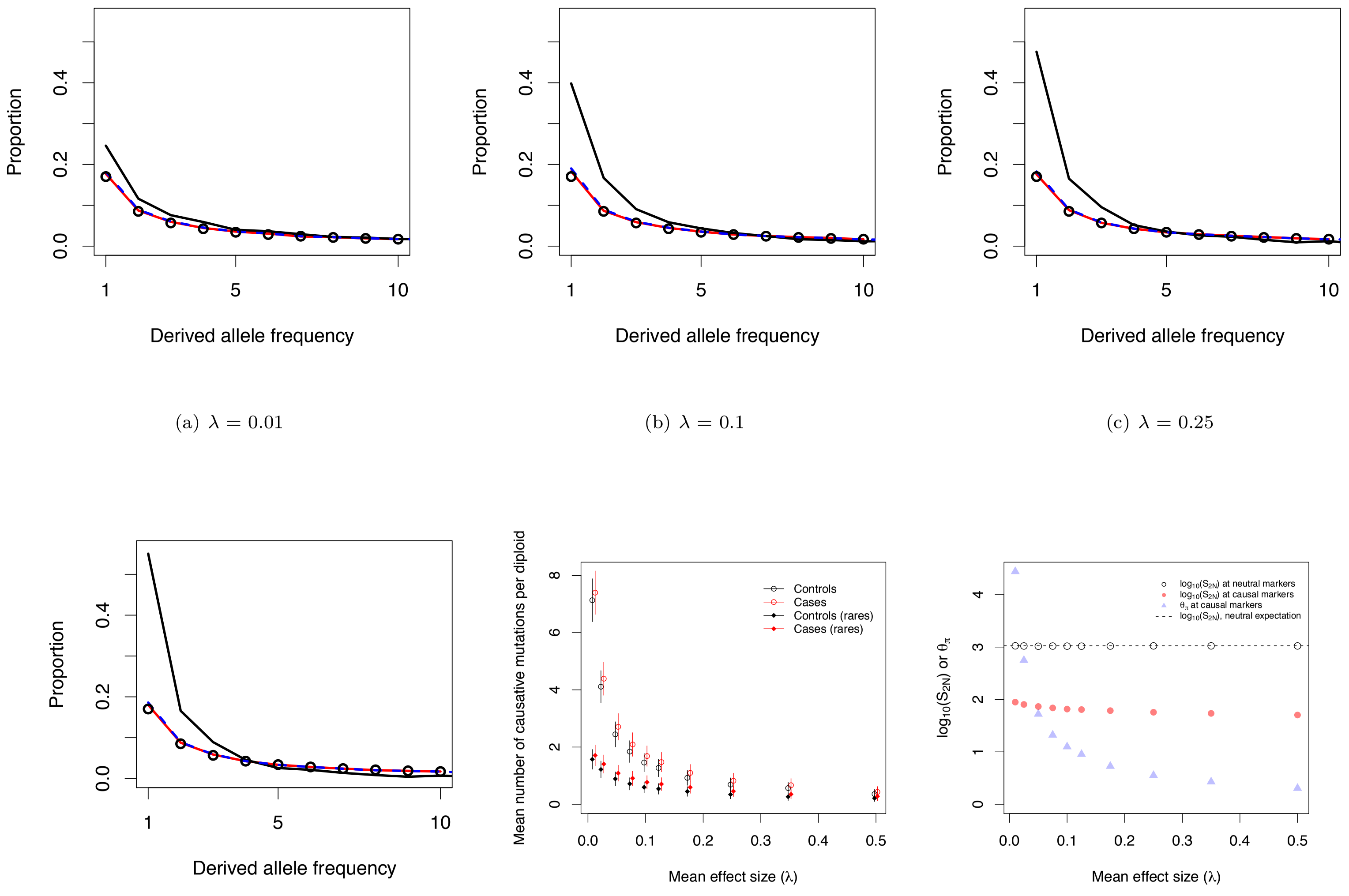

(a) $\lambda = 0.01$

(b) $\lambda = 0.1$

(c) $\lambda = 0.25$

(d) $\lambda = 0.5$

(e) Mean number of causative mutations per diploid $\pm\frac{1}{4}$ standard deviation

(f) Summaries of diversity in the population

Figure S3: Population-genetic properties of a locus.
