# Supplementary material for: Properties and Modeling of GWAS when Complex Disease Risk Is Due to Non-Complementing, Deleterious Mutations in Genes of Large Effect
Source: PLoS Genet. 2013 Feb 21;9(2):e1003258. doi: 10.1371/journal.pgen.1003258 (PMC3578756; doi:10.1371/journal.pgen.1003258)
Supplement: Figure S4 — Statistical properties of association studies. (a) Average proportion of rare variants which are causative in either the general population, in a case-control panel, or amongst significant markers in a GWAS where individuals are completely sequenced (b) For every neutral, common marker in a GWAS that was significant in a logistic regression test at p≤10−8, we measured LD using the r2 statistic between the significant marker and all causal markers in the case-control panel, and recorded the top two r2 values. The distribution of r2 for the top marker is summarized in white boxplots, and the distribution for r2 for the second-strongest association is summarized in red. (c) White boxes summarize the distribution of the number of significant, common, neutral markers, conditional on there being at least one such marker. The red boxes summarize the distribution of the number of unique causal markers amongst the top r2 values for each significant marker. Taken together, panels a and b suggest that significant common markers tend to tag a single causative site. (d) For each of the most strongly-tagged causal mutations making up the red boxes in panel b, the frequency and effect size of each mutant was recorded. The frequencies are summarized in the white boxes, and effect sizes are in red. (PDF) [file pgen.1003258.s004.pdf]

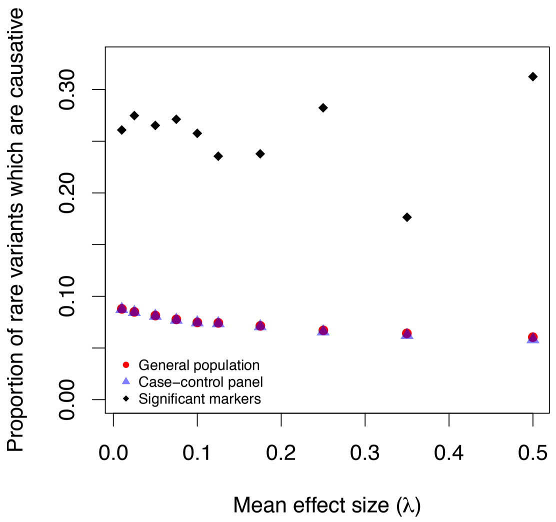

(a) Proportion of rare variants which are causative

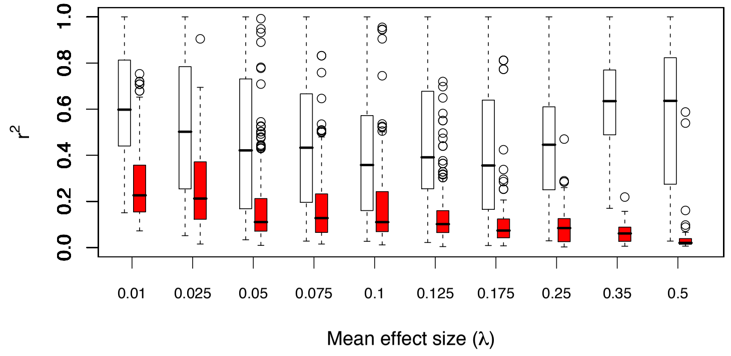

(b) Linkage disequilibrium between significant common markers and causal markers

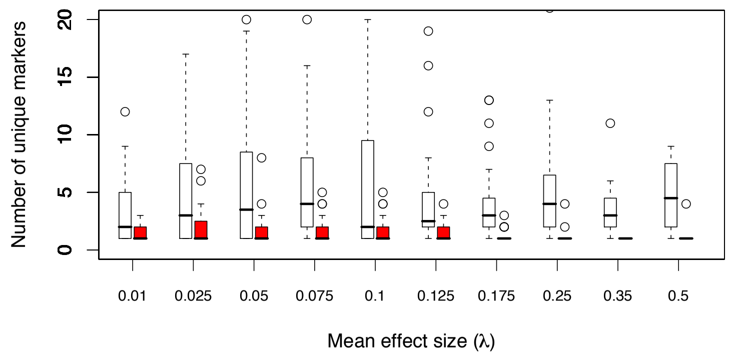

(c) The number of unique causal markers associated with significant common markers

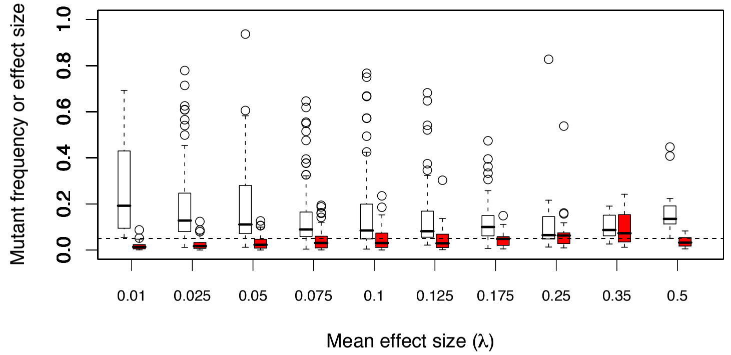

(d) Properties of causal markers associated with significant, common, neutral markers

Figure S4: Statistical properties of association studies.
