# Supplementary material for: Properties and Modeling of GWAS when Complex Disease Risk Is Due to Non-Complementing, Deleterious Mutations in Genes of Large Effect
Source: PLoS Genet. 2013 Feb 21;9(2):e1003258. doi: 10.1371/journal.pgen.1003258 (PMC3578756; doi:10.1371/journal.pgen.1003258)
Supplement: Figure S5 — Distributions of −log10 p-values for the Madsen and Browning [16] statistic. For all panels, the significance threshold of 10−6 is shown. (a) The empirical cumulative distribution function (ECDF) of p-values for control simulations with no deleterious alleles. As expected, the ECDF of p-values is a straight line with a slope of approximately 1. (b–k) ECDF of p-values for simulations with non-zero mean effect sizes of causative mutations (λ>0). (PDF) [file pgen.1003258.s005.pdf]

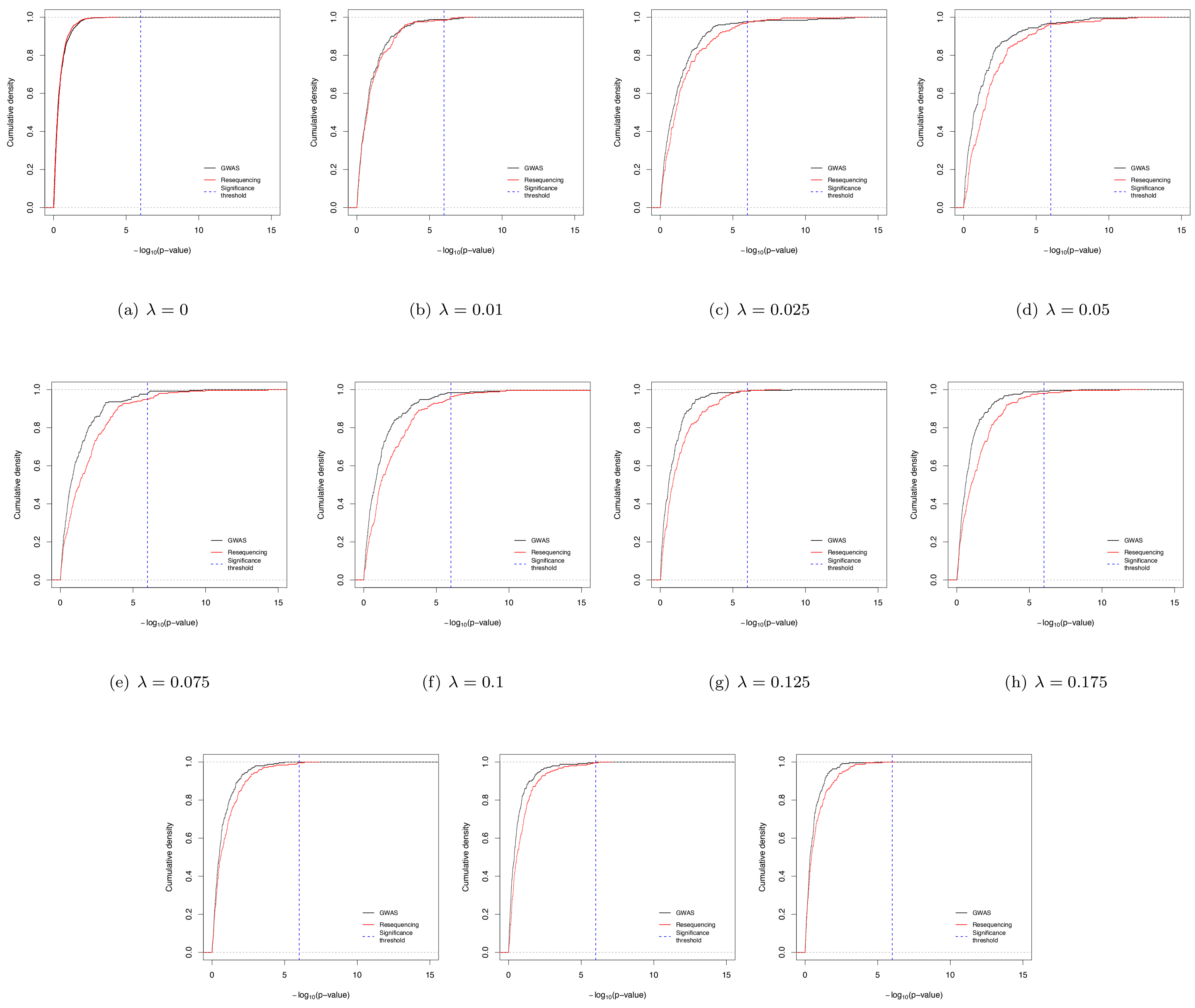

(a) $\lambda = 0$

(b) $\lambda = 0.01$

(c) $\lambda = 0.025$

(d) $\lambda = 0.05$

(e) $\lambda = 0.075$

(f) $\lambda = 0.1$

(g) $\lambda = 0.125$

(h) $\lambda = 0.175$

(i) $\lambda = 0.25$

(j) $\lambda = 0.35$

(k) $\lambda = 0.5$

Figure S5: Distributions of $-log_{10}$ p-values for the Madsen and Browning [4] statistic.
