# Supplementary material for: Properties and Modeling of GWAS when Complex Disease Risk Is Due to Non-Complementing, Deleterious Mutations in Genes of Large Effect
Source: PLoS Genet. 2013 Feb 21;9(2):e1003258. doi: 10.1371/journal.pgen.1003258 (PMC3578756; doi:10.1371/journal.pgen.1003258)
Supplement: Figure S10 — Distributions of −log10 p-values for the ESM statistic (see Methods) with no recombination. For all panels, the significance threshold of 10−6 is shown. (a) The empirical cumulative distribution function (ECDF) of p-values for control simulations with no deleterious alleles. As expected, the ECDF of p-values is a straight line with a slope of approximately 1. (b–k) ECDF of p-values for simulations with non-zero mean effect sizes of causative mutations (λ>0). (PDF) [file pgen.1003258.s010.pdf]

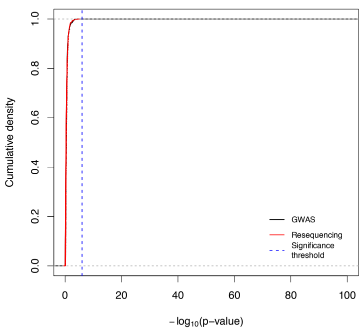


(a) $\lambda = 0$

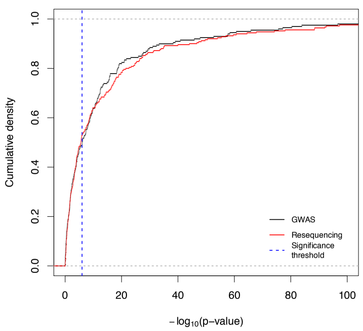


(b) $\lambda = 0.01$

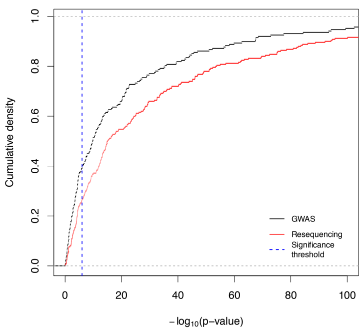


(c) $\lambda = 0.025$

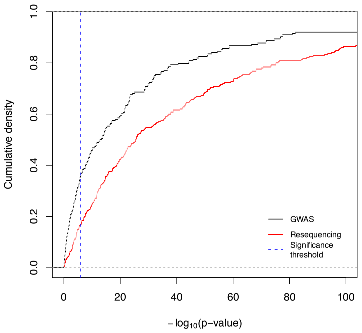


(d) $\lambda = 0.05$

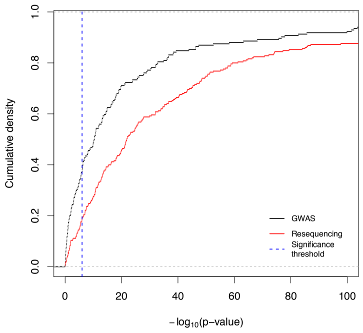


(e) $\lambda = 0.075$

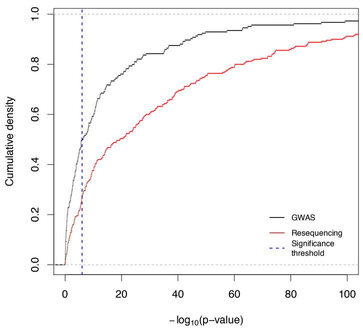


(f) $\lambda = 0.1$

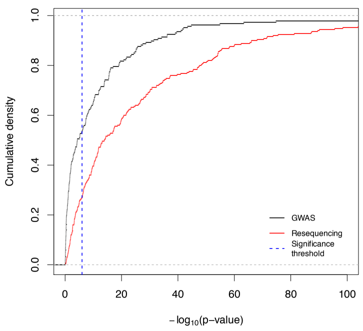


(g) $\lambda = 0.125$

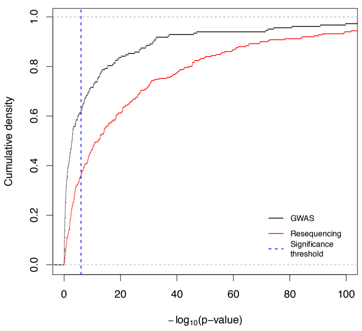


(h) $\lambda = 0.175$

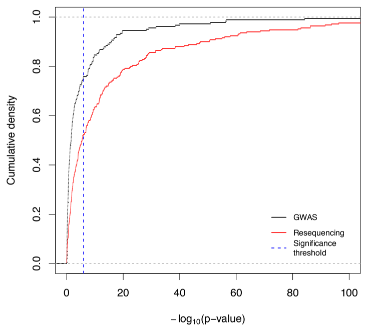


(i) $\lambda = 0.25$

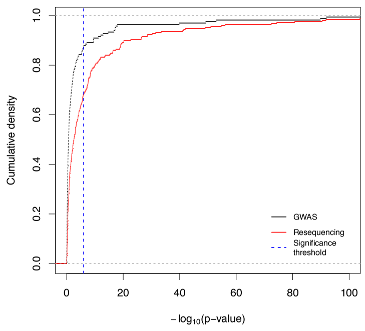


(j) $\lambda = 0.35$

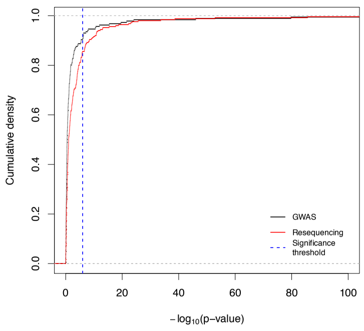


(k) $\lambda = 0.5$

Figure S10: Distributions of $-log_{10}$ p-values for the ESM statistic (see Methods) with no recombination.
