# Supplementary material for: Properties and Modeling of GWAS when Complex Disease Risk Is Due to Non-Complementing, Deleterious Mutations in Genes of Large Effect
Source: PLoS Genet. 2013 Feb 21;9(2):e1003258. doi: 10.1371/journal.pgen.1003258 (PMC3578756; doi:10.1371/journal.pgen.1003258)
Supplement: Figure S11 — A comparison of the burden of risk mutations between significant and non-significant markers in a GWAS. Shown are mean and standard errors of the number of causative singletons in individuals with genotypes defined as having either zero, one, or two copies of the derived allele at the marker most significantly associated (e.g., the smallest p-value) with case/control status in a GWAS analyzed by a single-marker test (red). Pale blue lines show the mean and standard errors of the number of causative singletons associated with zero, one, or two copies of the derived mutation of a marker that is both not associated with case/control status in a logistic regression analysis (p>10−4) and frequency-matched to the most-associated marker in the same replicate. Each panel of the figure is labeled by the mean effect size of a causative mutation (λ). (PDF) [file pgen.1003258.s011.pdf]

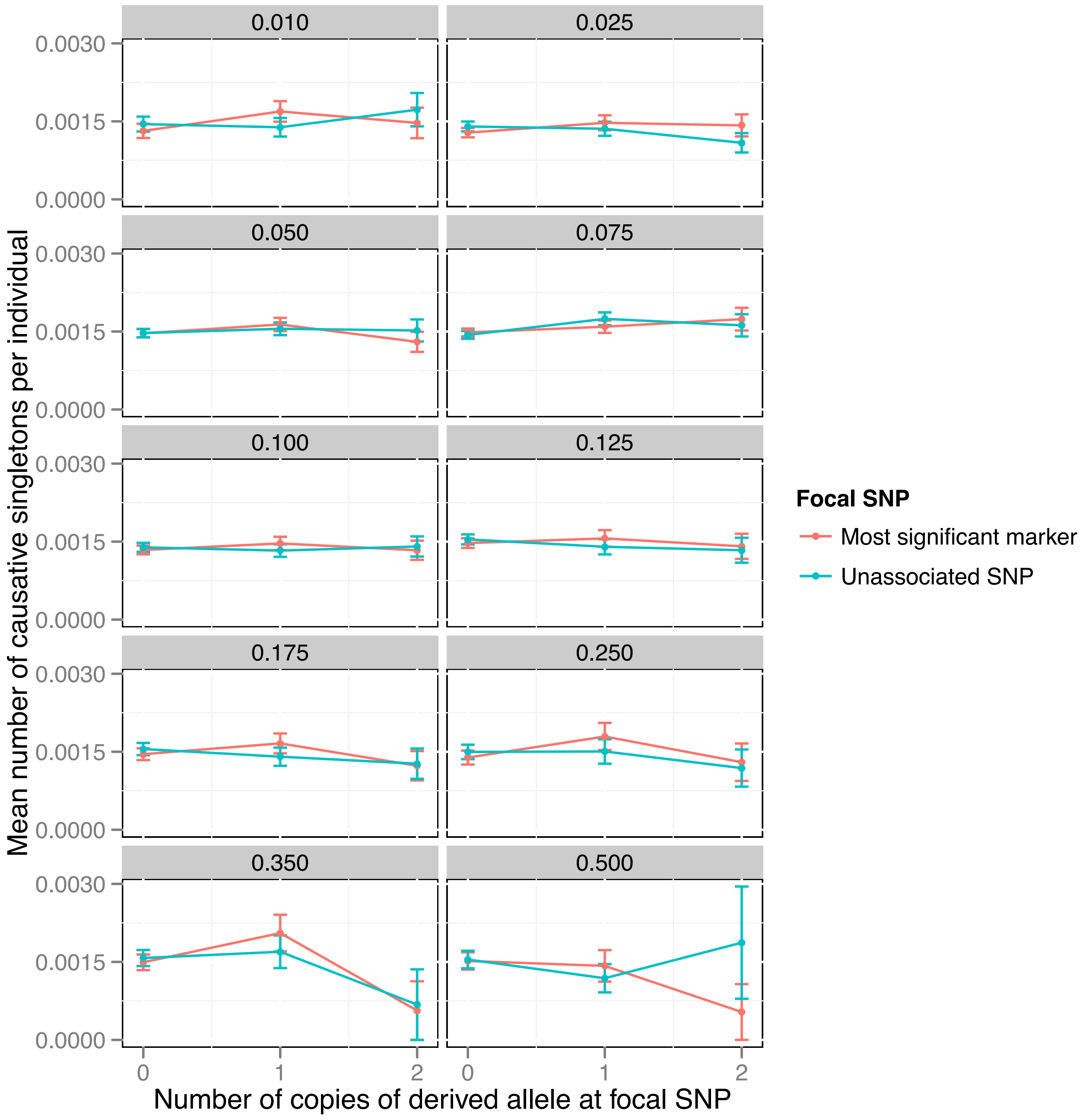

Figure S11: A comparison of the burden of risk mutations between significant and non-significant markers in a GWAS.
